# Pathways to High-Performance Salt Hydrate Thermochemical Energy Storage Materials and Systems

**DOI:** 10.1021/acsenergylett.5c03249

**Published:** 2026-01-09

**Authors:** Madeline R. Morrell, Srivatsa Bhat Kaudur, Jungho Shin, Sadie Flagg, Ishita Goyal, Jaechan Pyo, Erik Barbosa, Satyam Bharti, Claudio V. Di Leo, Matthew T. McDowell, Akanksha K. Menon

**Affiliations:** † G. W. Woodruff School of Mechanical Engineering, 1372 Georgia Institute of Technology, Atlanta, Georgia 30332, United States; ‡ Daniel Guggenheim School of Aerospace Engineering, 1372 Georgia Institute of Technology, Atlanta, Georgia 30332, United States; § School of Materials Science and Engineering,Georgia Institute of Technology,Atlanta, Georgia 30332, United States

## Abstract

Thermochemical materials (TCMs) based on salt hydrates are promising for thermal energy storage as they combine high energy densities with low reaction temperatures. However, their adoption is hindered by poor structural integrity and degradation under hygrothermal cycling. Storage performance is governed not only by the chemical reaction, but also by the coupled thermo-chemo-mechanical behavior that evolves with cycling. Understanding and controlling this coupling across length scales (material-to-reactor) is necessary to improve TCM stability and lifetime. In this perspective, we discuss the shortcomings of current characterization approaches and emphasize the need for measuring transport properties and structural transformations using *in situ* techniques that capture the dynamic evolution of these materials. We also outline opportunities for multiscale modeling frameworks that link thermodynamics and mechanics, enabling predictive evaluation of composite architectures designed for cycling stability. We conclude by identifying research questions that must be addressed to transform TCMs into viable energy storage technologies.

## Graphical abstract

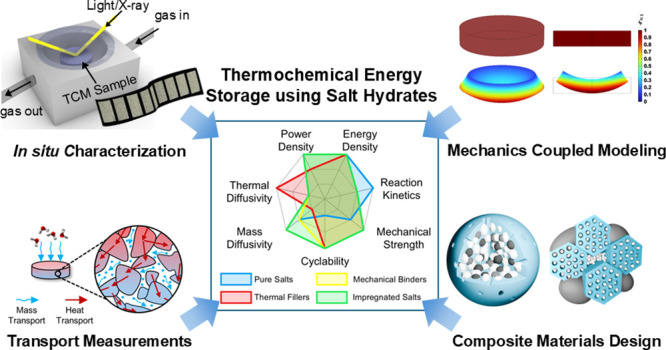

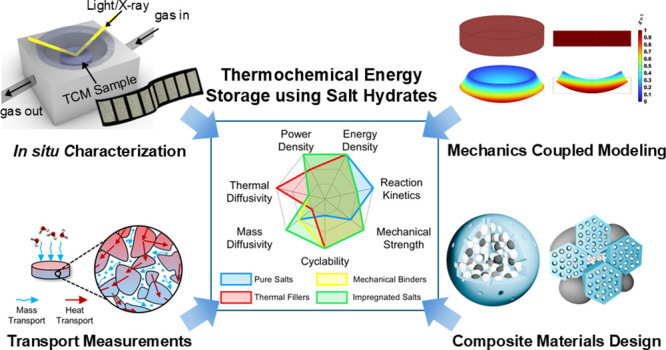

Heat is the dominant form of energy end-use globally, with 90% of primary energy being associated with its generation and conversion across a range of temperatures for industrial processes and buildings.^1,2^ The buildings sector alone consumes a significant portion of the world’s energy, with half of this being used to meet thermal loads (heating/cooling).^3,4^ Most of this energy is produced from fossil fuel combustion, resulting in significant carbon emissions.^2^ The transition to a decarbonized energy sector with increased penetration of renewables requires cost-effective energy storage at the TWh-scale to address intermittency and enable dispatchability. Significant research and development for over a century has focused on electrochemical batteries, but this form of storage is best suited for rapid charge–discharge cycles over short durations (<4 h). Furthermore, when the end-use is in the form of heat instead of electricity (as is the case for buildings and industry), additional energy conversion steps can result in losses and higher cost.^5^ In this scenario, thermal energy storage (TES) is promising for storing energy (heat and electricity) for peak shifting and shaving of thermal loads over longer durations (>10 h). Specifically, TES can function as a thermal battery by decoupling power density and energy density through careful design of the material (e.g., thermal conductivity) and system (e.g., cell geometry).^6^ In comparison to electrochemical batteries, TES also benefits from leveraging abundant and low-cost materials with negligible losses during long-term storage.^7,8^

TES can be separated into three categories: sensible, latent (phase change), and thermochemical heat storage. Among these, thermochemical materials (TCMs) that undergo reversible endothermic-exothermic reactions are arguably the most desirable as they exhibit relatively high energy storage densities and negligible self-discharge. The reaction can occur at different temperatures depending on the solid–gas pair, allowing for a wide range of use-cases from concentrated solar power and industrial process heat (∼800 °C),^9–11^ to space conditioning and domestic hot water (<100 °C).^7,12^ TCMs are often categorized as sorbents, which are relevant for applications beyond TES including metal hydrides for hydrogen storage,^13^ carbonates for carbon capture,^14^ metal organic frameworks (MOFs) for catalysis,^15^ and salt hydrates for dehumidification.^16^

For the commercial and residential buildings sector, which accounts for more than a third of the primary energy consumption in the United States,^17^ salt hydrates are suitable TCMs owing to their nontoxic nature and low charge/discharge temperatures.^18^ Specifically, salts such as MgSO_4_, SrBr_2_, SrCl_2_, K_2_CO_3_, CaCl_2_, and MgCl_2_ have been explored due to their high energy storage density (>500 kWh/m^3^) and suitable charge–discharge temperatures (<100 °C).^7,18,19^ In this case, the salt (solid) reacts with water vapor (gas) to transition from one hydrated phase to another, as shown by the equilibrium reaction (eq 1):

$$\mathrm{Salt}\cdot aH_{2}O+\Delta H⇌\mathrm{Salt}\cdot bH_{2}O+\left(a-b\right)H_{2}O$$ 1

The high energy storage density arises from a large reaction enthalpy (Δ*H*) and number of moles of water exchanged (*a-b*). The enthalpy and entropy (Δ*S*) of these reactions can be predicted using density functional theory (DFT), accounting for changes in crystal structure. The thermodynamic conditions governing hydration and dehydration, specifically vapor pressure and temperature (hygrothermal parameters), are then determined through the Clausius–Clapeyron relation which can be illustrated with phase diagrams that depict each salt and its stable hydrated phases.^20^ Despite their promising theoretical performance, fundamental advances are necessary to enable their practical implementation and long-term stability for TES. Specifically, the reversibility of the reaction during continuous charge/discharge cycling is limited by several coupled factors.^21^ For example, chemo-mechanical challenges arise from volumetric expansion/contraction of the salt as it accommodates water vapor during hydration/dehydration.^22,23^ These transformations and the associated stress evolution are not uniform owing to the propagation of a reaction front, as well as the presence of thermal/concentration gradients within the material. This leads to microstructural evolution and macroscale mechanical degradation, which diminishes transport and storage capacity over a few cycles.^24^ In the field of electrochemical batteries, a recent focus on understanding the coupled electro-chemo-mechanics has resulted in design paradigms for improving electrode design and cycle life of these systems.^25,26^ Similarly, the foundational framework to understand the coupled thermo-chemo-mechanics in salt hydrate TCMs needs to be developed. This perspective outlines the fundamental thermochemical and transport properties, characterization techniques, continuum models, and material design strategies that can unlock the knowledge required to improve TCM performance. Finally, we outline the open research questions that are necessary to bridge existing scientific gaps and enable successful implementation of TCM-based energy storage.

## Material Property Evolution with Cycling

The first step toward enabling high-performance thermochemical energy storage systems is material property characterization for these coupled systems. Thermochemical properties (e.g., reaction enthalpy and kinetics) along with thermophysical properties (e.g., thermal conductivity, specific heat, and diffusion coefficients) are both necessary to develop heat and mass transport models that are coupled with reaction kinetics. However, there are discrepancies in how these properties are characterized in literature owing to the lack of standardized approaches. Additionally, these properties evolve with hygrothermal cycling owing to mechanical changes (e.g., volume). This signifies the need for standardized techniques that can capture the dynamic behavior of these materials over their lifetime, which is the focus of this section.

### Storage Capacity and Reaction Kinetics

Energy and power density of TCMs depend on coupled mass and heat transport, thermophysical properties, reaction kinetics, and mechanical properties – all of which impact cycling stability. Figure 1A shows the different desired characteristics for a high-performance thermal battery, and the status of current pristine salt hydrates. These properties govern overall thermo-chemo-mechanical behavior, and they must be properly characterized so that accurate models can be developed to predict system-level performance (e.g., in a packed bed reactor), as we discuss in this perspective.

**Figure 1:**
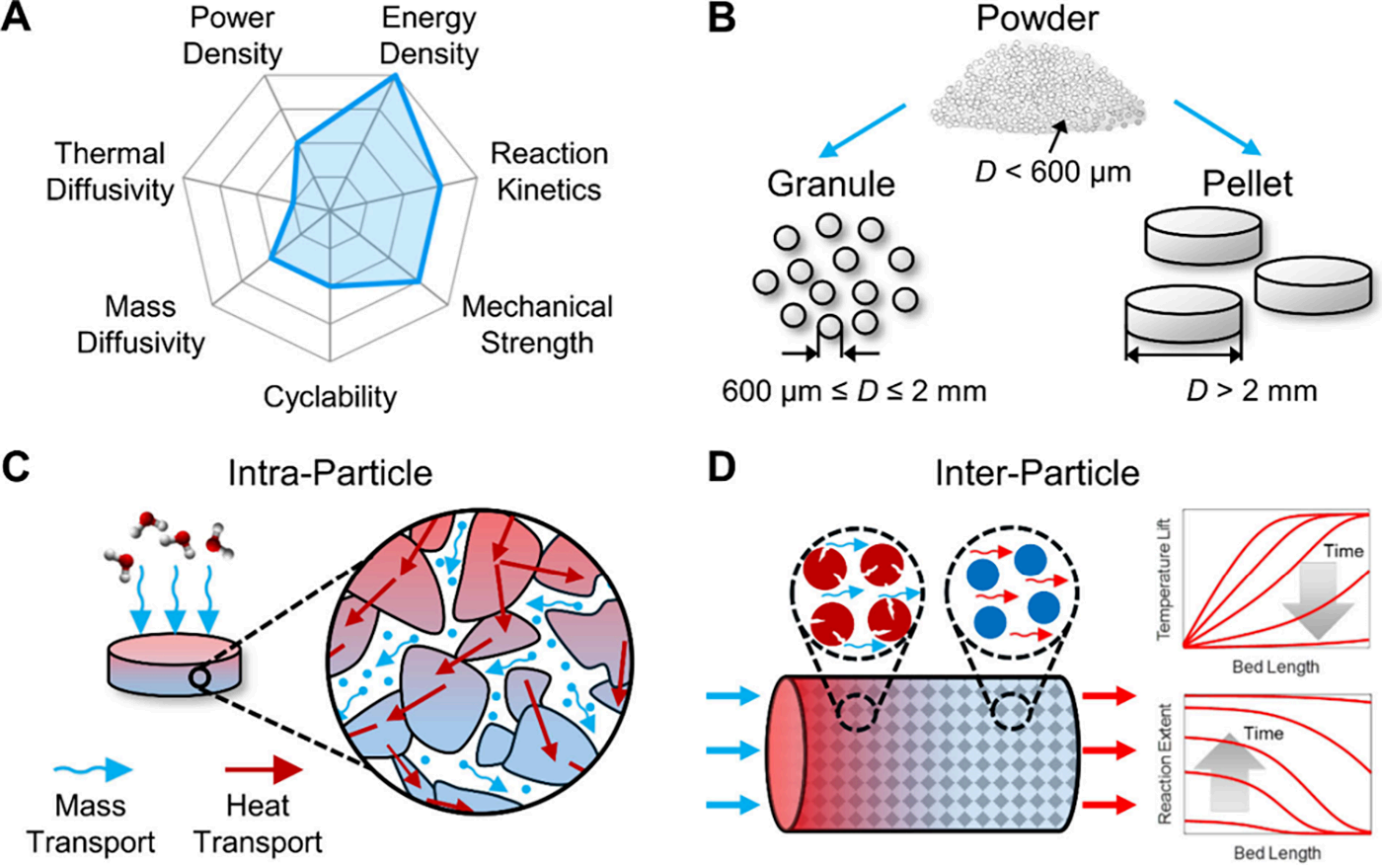
Performance of salt hydrate-based energy storage is governed by coupled thermo-chemo-mechanics: (A) Summary of desired characteristics for a thermal battery and the properties of pristine salt hydrates that have been reported in the literature. (B) Material form factor affects coupled heat and mass transport by changing the resistance to flow. Transport occurs at two length scales, including (C) within a particle (intraparticle) and (D) between particles (interparticle) within a packed bed reactor system. During hydration, water molecules diffuse through the length of the bed to induce the exothermic chemical reaction, and the heat generated by the salts can be transferred to the air.

Energy and power density of TCMs depend on coupled mass and heat transport, thermophysical properties, reaction kinetics, and mechanical properties – all of which evolve with and impact cycling stability.

Simultaneous thermogravimetric analysis and differential scanning calorimetry (TGA/DSC) is commonly utilized to screen and down-select salt hydrates based on their reaction enthalpy (energy storage density).^7,12,27^ However, significant variations exist in measured values for the same material owing to differences in experimental parameters used, such as sample mass, heating rates, and reactive gas flow rate.^28^ For example, the energy density of CaCl_2_·6H_2_O was reported to be 801 J/g and 2203 J/g in two different studies,^29,30^ while the theoretical value for this salt is 2280 J/g.^27^ These variations arise because thermodynamically favorable reactions may be hindered by kinetic limitations such as nucleation barriers or metastable zones.^31^ Additionally, hygrothermal instabilities can hinder vapor and thermal transport within salt hydrates. For instance, excess water vapor during the hydration reaction can cause the salt to form a liquid solution (deliquescence). In addition to hygrothermal instabilities, the low thermal conductivity of these salts can cause the salt hydrate to melt during the dehydration reaction that occurs at higher temperatures. These liquid phases or agglomerates can severely inhibit the diffusion of vapor through the salt microstructure, leading to slow or incomplete reactions that manifest as reduced energy and power densities.^32^ The energy and power density of TCMs should thus be characterized under carefully selected hygrothermal conditions guided by phase diagrams that include these considerations. Furthermore, the cycling performance should be measured in a TGA/DSC system that is integrated with a humidity generator to minimize material exposure to the lab environment during hydration/dehydration.

TGA/DSC is also used to capture reaction kinetics, which describe the rate at which salt reacts with water vapor. Hydration kinetics normally rely on a reaction rate constant, which can be characterized by measuring the initial reaction rate of a small sample (∼1 mg) of salt before diffusion effects begin to slow the process. Fisher et al. used TGA/DSC data to compare different reaction models (e.g., nucleation, diffusion, and reaction-order models) and obtained rate constants using differential and linear fits for salt hydrates and composites at varying temperatures.^33^ It is important to note, however, that the reaction rate constants that are measured at specific hydration/dehydration conditions are only valid for those cases. This suggests the need for a database of Arrhenius-type kinetic constants, which would minimize the number of experiments needed to extract reaction rates over a range of conditions.

### Transport Properties

Many studies have screened TCMs based on their thermochemical properties characterized using TGA/DSC.^7,27,33^ The corresponding energy density is commonly reported^7,18^ as the primary metric for salt selection, but power density is equally important from an application standpoint and has not been well characterized. This is in part because thermal power output is sensitive to the system geometry and fluid coupling, with packed bed reactors being the most common design for salt hydrate-based TES. At the material level, however, thermophysical and transport properties (e.g., thermal conductivity and diffusion coefficients) influence thermal power output.

Thermal diffusivity quantifies the rate at which heat propagates through the material, which is important because the exothermic hydration reaction generates heat that must be extracted from the packed bed reactor. Thermal diffusivity can be measured using laser flash analysis, which can be coupled with specific heat measurements obtained from a DSC via the dynamic method,^34^ to calculate thermal conductivity.^35^ A number of different techniques have been used to report thermal conductivity values, including DSC, guarded hot plate/cartridge, heat flow meter, transient hot wire, and modified transient plane source.^36–38^ However, the effective thermal conductivity of salt hydrates is low (<1 W/m-K), resulting in substantial differences between measurement techniques.^39^ For instance, high void fractions and poor thermal contact can result in variations of the effective thermal conductivity. An additional challenge is with reactions (phase transitions) occurring during the measurement due to exposure to the lab environment. Obtaining accurate specific heat data has also proven difficult due to the challenge of maintaining the same hydrated phase throughout the measurement, as recent round-robin studies have shown.^40^ This suggests the need to perform specific heat measurements using an actively cooled DSC that allows for the hydrated phase to remain stable over the temperature ramp of the measurement. This has been demonstrated with epsomite (MgSO_4_·7H_2_O) at cryogenic conditions,^41^ and can be extended to other hydrated salts.

The mass diffusivity or diffusion coefficient provides a measure of the mobility of water vapor through the packed bed. Often, this diffusion coefficient is approximated as the product of porosity and the diffusion coefficient of water vapor in air (since the diffusion coefficient of vapor within the salt is orders of magnitude lower). This simplification, however, does not account for the tortuosity of the network and assumes that parallel vapor diffusion pathways exist in the material. This in turn does not capture the real vapor diffusion rate as the structure of the porous network varies depending on the material and evolves with hygrothermal cycling. Instead, the wet cup technique can be used to measure mass diffusivity by maintaining a vapor pressure driving force across the salt.^24^ This approach has been demonstrated with K_2_CO_3_·1.5H_2_O,^24,42^ but may be limited for other salts owing to challenges with identifying suitable desiccants that have low saturation relative humidities to maintain the hydrate phase.^27,43^

It is also important to note that these transport properties are highly dependent on material microstructure (porosity and tortuosity) and form factor, which can be categorized as powders, granules, or pelletized particles, as shown in Figure 1B. Powder is generally processed via ball-milling and sieving, and it can be formed into granules or pellets with wet granulation or a hydraulic press. These form factors correspond to multiple length scales for transport, which results in different effective properties and reaction time scales. Intraparticle transport is concerned with heat and mass transfer occurring within a salt particle (Figure 1C), while interparticle transport captures the heat and mass transfer between particles within a packed bed system (Figure 1D). Thermal transport predominantly occurs at grain boundaries within the solid salt and between interconnected salt particles in the reactor given their higher thermal conductivity compared to air. Mass transport, on the other hand, occurs as water vapor travels through the voids of the salt crystal and between particles that form a porous network in the reactor. Varying the material microstructure and form factor can thus significantly impact the measured transport properties, especially in the pelletized form factor where vapor diffusion limits reaction advancement.^24^ This suggests the need to move from powders to pellet characterization, as this form factor is relevant for use in a reactor.^23^

### Degradation Mechanisms

Transport pathways are also altered by repeated cycling, which can accelerate mechanical degradation and lead to variability in energy storage performance. Stepwise phase transformations generate large intrinsic lattice expansions or contractions, bond rearrangements, and structural reconfigurations at the atomic level. Since these transformations likely proceed along vapor diffusion pathways, they are path-dependent and nonuniform within the material. The resulting strain mismatch – intensified by propagating reaction fronts – concentrates stress and creates preferential failure paths at the microscale. Additionally, microstructural features (e.g., porosity, tortuosity, particle size, etc.) can serve as stress concentrators that induce local stresses during repeated cycling that manifest as cracks. This is because the material expands/contracts substantially to accommodate water molecules into the crystal structure, creating nonuniform mechanical strains within the microstructure.^22,23,44^ For example, SrCl_2_ can undergo an ∼165% theoretical expansion in volume between its dehydrated and hydrated phases, which in turn causes mechanical stress concentration and crack initiation.^22^ Aarts et al. also showed that hygrothermal cycling induces swelling, cracking, and a more tortuous or isolated pore network in K_2_CO_3_ pellets.^24^ Ultimately, stress generation results in pulverization of salt particles at the macroscale. Martin et al. developed a model to predict the pulverization limit (*R*_crit_) for salt hydrate powders during cycling, and they examined how this threshold influences reaction kinetics.^45^

A holistic understanding of mechanical degradation mechanisms in salt hydrates remains limited. Most studies to date have relied on *ex situ* analysis: materials are cycled under controlled temperature and vapor pressure (relative humidity), and only the “before” and “after” phases are characterized. This approach is useful for collecting data once damage has already accumulated; examples include fragmentation, porosity loss, and grain coarsening. However, *ex situ* snapshots miss the transient evolution that governs how mechanical damage arises and propagates. As a result, the true rate-limiting steps in complex reactions with multiple stable intermediary hydrates (e.g., in SrCl_2_, CaCl_2_) can be misidentified when only end states are examined. This limitation has been increasingly highlighted in recent reviews calling for time-resolved, *in situ* characterization to reveal transport and phase transition pathways.^22,46,47^ Micro-CT results^48^ underscore this gap clearly: after ten cycles, the porosity of a packed bed decreased significantly from ∼41% to ∼20% with an increase in grain size of the salt, yet the sequence and drivers of these changes during each cycle remain invisible without time-resolved data. Additionally, *ex situ* snapshots rarely observe concentration gradients that arise as the reaction front moves through pellets within a packed bed. Without this information, it is difficult to attribute damage to a specific phase transition or to rate-dependent evolutions, especially in complex salt hydrates (e.g., SrCl_2_) where the intermediate hydrated phases appear during cycling.^48,49^

### *In Situ* Characterization

*In situ* characterization can be used to observe the structural and morphological evolution of materials under realistic operating conditions. In the field of electrochemical batteries, *in situ* characterization has been used to resolve the coupled evolution of structure, chemistry, and mechanical stress under relevant conditions.^50–53^ Real-time characterization, such as X-ray diffraction (XRD), X-ray tomography (XCT), optical microscopy with digital image correlation (DIC), Raman spectroscopy, and mechanical stress measurements, have provided fundamental insight into the evolution of battery materials. These methods can delineate intercalation/conversion fronts, transient intermediates, lattice-mismatch strains, interface damage, and crack initiation-coalescence-propagation. This in turn enables a link between structural transitions and morphological response, thereby enabling the rational design of mechanically robust batteries. This can be extended to salt hydrate TCMs with an analogy: water vapor diffusion and temperature fields evolve in space and time, drive phase transformations with significant mechanical deformations, and generate strain mismatch-induced stresses due to nonuniform volume changes. As such, a synchronized, multimodal *in situ* framework for TCMs can be used to (i) visualize operating regimes under realistic conditions, including temperature ramp rates and relative humidity variations, (ii) quantify front kinetics and transient intermediate phases, and (iii) establish couplings between thermodynamic driving forces, structural transitions, transport bottlenecks, and mechanical damage.

To enable *in situ* characterization of TCMs, experimental cells must be purpose-built for transmission of both the interrogating and emitted signals, and they must be equipped with environmental control, as shown in Figure 2A. Transmissive windows (e.g., sapphire or glass/Kapton for optical/Raman access and thin Al/Be or Kapton for X-rays) should be precisely aligned and hermetically sealed to provide colinear beam paths, sufficient field of view, and resistance to humid/halide environments. With synchronized timing across sensors, a custom *in situ* thermochemical cell can support multimodal interrogation, directly linking structural transitions, transport, and stress under realistic operating conditions, as shown in Figure 2B and 2C.^22^ For example, recent work has combined *in situ* optical microscopy (capturing crack initiation/propagation and volume changes) with *in situ* XRD (resolving phase-fraction kinetics, transient hydrates, and lattice strain), which provided insight into the mechanisms by which cycling induces mechanical stress and drives cracking in SrCl_2_ (Figure 2C).^22^ *In situ* X-ray computed tomography (XCT) may be particularly valuable for observing chemo-mechanical degradation in TCMs (Figure 2C).^54–56^ By providing volumetric, time-resolved 3D image data sets of TCMs, *in situ* XCT can visualize crack nucleation and propagation, pore formation/growth/closure, particle fragmentation, and reaction front curvature, which are inaccessible via surface or 2D imaging methods. The dynamic tracking of (de)­hydration reaction in salt hydrates using *in situ* techniques could enable an understanding of the rate-limiting steps and effect of structural evolution on bulk transport properties. Correlating to other measurements (e.g., *in situ* microscopy, *in situ* XRD, TGA/DSC) would enable causal attribution of damage to specific kinetic regimes and to particular phase transitions. These insights, in turn, would guide material design and selection, as well as operating protocols that reduce mechanical stress concentration and avoid damage.

**Figure 2:**
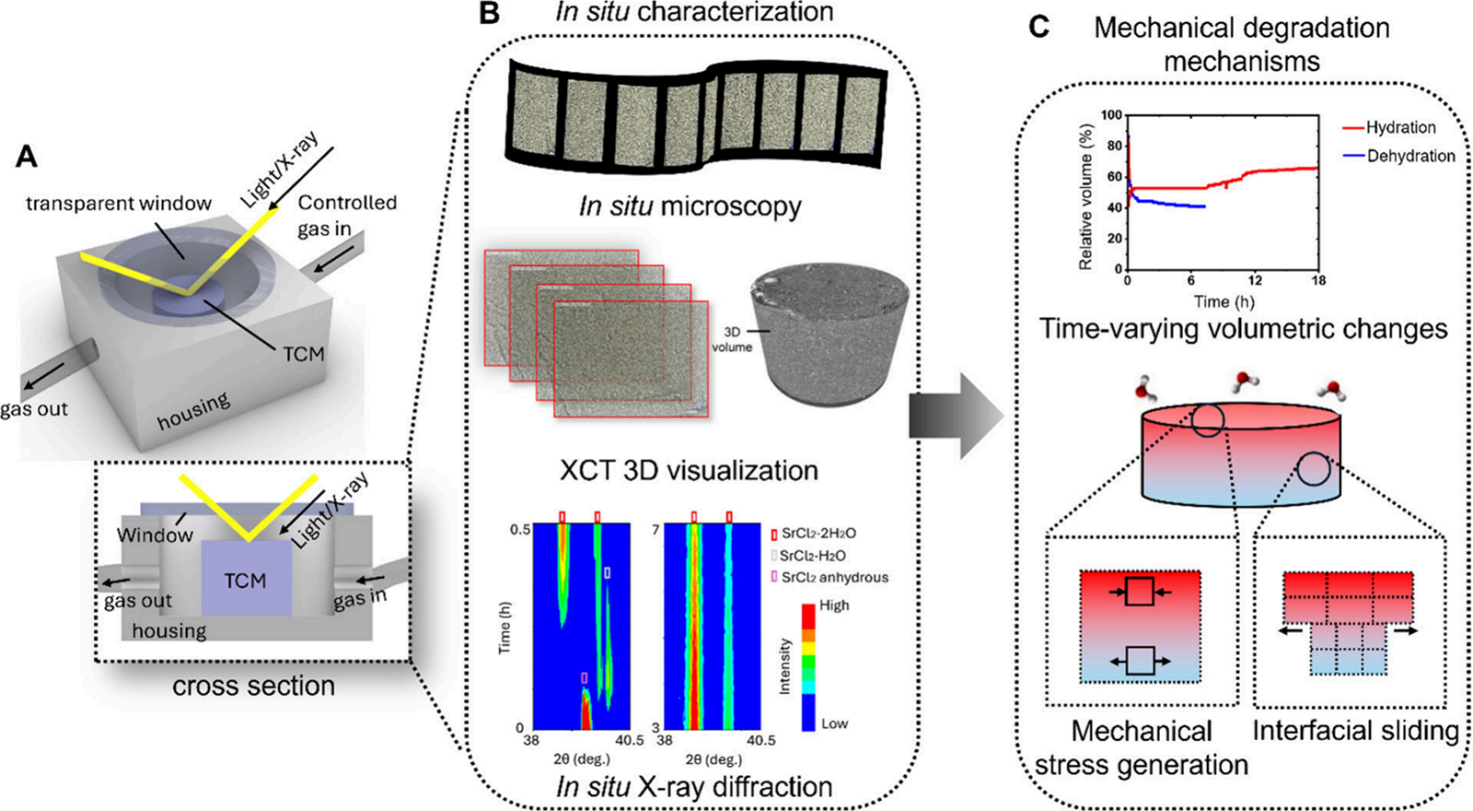
*In situ* characterization of TCMs to capture their dynamic evolution. (A) Schematic of the *in situ* testing setup for real-time monitoring of TCMs during hydration–dehydration cycling. (B) Optical- or X-ray-transparent windows allow either optical microscopy or X-ray measurements (XRD/XCT). (C) Time-aligned data sets correlate phase evolution and front propagation with cracking, swelling/shrinkage, and delamination, revealing mechanical degradation mechanisms. (B) and (C) are adapted with permission from the Royal Society of Chemistry.^22^

The dynamic tracking of (de)­hydration reaction in salt hydrates using *in situ* techniques could enable an understanding of the rate-limiting steps and effect of structural evolution on bulk transport properties.

## Modeling Coupled Thermo-Chemo-Mechanical Systems

Standardized and real-time characterization serves as the foundation to develop robust thermo-chemo-mechanical models for predicting system-level performance. This section details the specific modeling approaches that can be used for TCM systems.

### Coupling Mechanics

Given that many of the degradation mechanisms manifest through mechanical effects, integrating mechanics at different length scales into TES models is essential for accurate prediction of material stability and system performance over its lifetime. Mechanics couples to chemistry across every relevant length scale, from the crystal lattice to the packed bed reactor, primarily by shifting local chemical equilibria, inducing deformations that alter the material form factor and system geometry, and modifying transport properties. Figure 3A illustrates the complex, fully coupled nature of a thermo-chemo-mechanical system with three primary two-way interactions: (1) temperature affects chemical equilibrium and reaction kinetics, (2) reaction front advancement produces nonuniform deformations, and (3) the resulting elastic stress fields feed back to the chemistry by modifying local chemical affinity and kinetics. The irreversible mechanical work associated with reaction-induced deformation appears as a source of thermal energy, so that dissipation modifies the local temperature field and therefore the subsequent chemical dynamics. The schematic thus emphasizes that thermal, chemical, and mechanical fields form a closed feedback loop.

**Figure 3:**
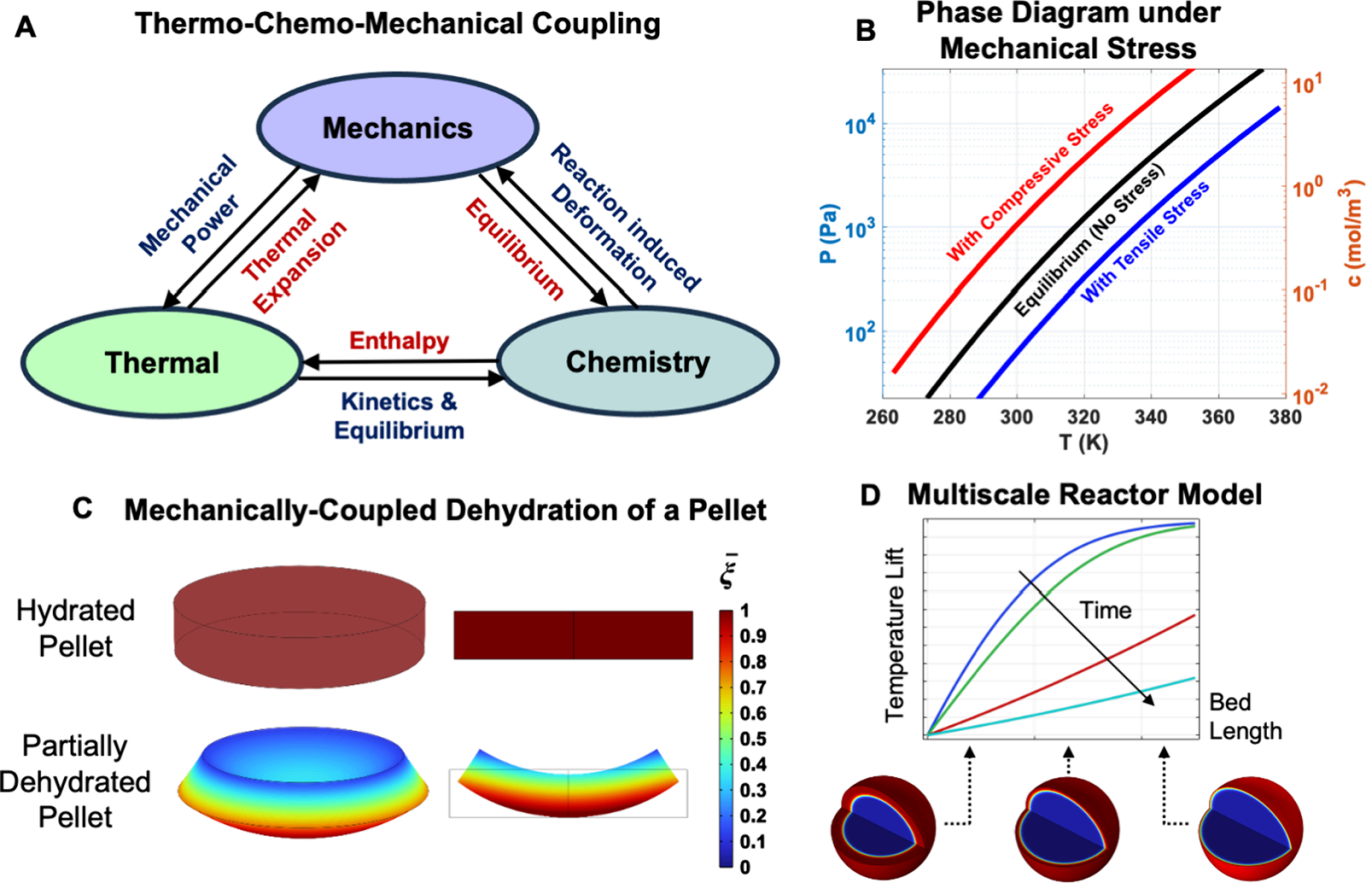
Fully coupled thermo-chemo-mechanical interactions that govern solid–gas reactions. (A) Schematic showing bidirectional couplings between thermal, chemical, and mechanical fields that define the behavior of a TCM-based system. (B) The result of compressive/tensile internal stresses on the phase diagram, altering the effective reaction affinity and perturbing the local chemical equilibrium, thereby accelerating or decelerating the reaction rate. (C) Representative results showing the deformations and modified reaction front morphology within a pellet form factor. (D) Multiscale reactor model where porous models are coupled to local fully resolved thermo-chemo-mechanical models of individual particles of TCMs. (C,D) The color map and contours correspond to reaction extent.

Given that many of the degradation mechanisms manifest through mechanical effects, integrating mechanics into TES models at different length scales is essential for accurate prediction of material stability and system performance over its lifetime.

The phase diagram in Figure 3B shows how stress shifts phase boundaries. Compressive stresses favor dehydration while tensile stresses favor hydration, so a given temperature can yield different local equilibria depending on stress. The reaction contours in Figure 3C demonstrate the mechanical consequences for a dehydration reaction. Reaction-induced deformations produce bending and heterogeneous stress fields that in turn bias local reaction progress, producing asymmetric dehydration fronts. Because inelastic work feeds back into the heat balance, the net input temperature required for dehydration (and the output temperature obtained during hydration) is altered relative to purely thermochemical predictions. Together, this shows that mechanics is an active control parameter for both thermodynamics and macroscopic reaction kinetics.^57^ Other mechanics-driven processes like pore closure or expansion, microcrack nucleation and healing, and the consequent changes in transport are not considered. Incorporating them would further alter front morphology and reaction rates.

### Modeling across Length Scales

The atomic-scale origin of thermo-chemo-mechanical coupling, i.e., how mechanical strain influences the energy barriers of reaction pathways, can be obtained using density functional theory (DFT)^58^ to calculate energy landscapes or molecular dynamics (MD) and reactive force fields (ReaxFF)^59^ to simulate the dynamic process of bond breaking and formation due to phase transformations and strain. Furthermore, MD and ReaxFF have shown the potential to predict the thermophysical properties during hydration-dehydration reactions, such as vapor diffusion coefficients, due to their ability to model crack propagation and porosity evolution.^60^ While these methods provide invaluable fundamental insight, they are severely limited by their short time scales (picoseconds to nanoseconds) and small length scales (nanometers), creating a significant gap to microscale experiments and pellet-scale behavior.

The micrometer to centimeter scale is where the interplay between reaction, diffusion, and deformation becomes apparent. It is small enough to avoid the extreme heterogeneity of a full reactor but large enough to exhibit the critical gradients that drive coupling. A continuum modeling approach is most effective here, solving the coupled partial differential equations for species (water vapor) transport, heat transfer, chemical kinetics, and solid mechanics within a finite element method (FEM) framework. The work by Kaudur and Di Leo^57^ is the first for TES systems that focuses on this full coupling, which is essential to capture stress-dependent morphology changes of the reaction front. This scale is also ideal for validating the fully coupled model against experimental techniques like *in situ* XCT, which can visualize the internal reaction front progression and crack formation, as discussed in the previous section.

Scaling up to a packed bed reactor, which consists of thousands of particles (powders, granules, or pellets), introduces the challenge of representing the mechanics of the entire aggregate. Two important methods are noted. First, in porous continuum models the reactor bed is treated as a single, effective porous medium. The model solves porous-medium transport of heat and mass, with a local reaction source term that must be calibrated from single-particle or pellet kinetics experiments (e.g., TGA).^61^ Mechanics can be incorporated in a homogenized sense, e.g., by defining a strain for the homogenized material or using continuum damage mechanics to model the average loss of stiffness and permeability over cycles. This approach is computationally efficient and suitable for engineering design and reactor-scale optimization but are of lower fidelity. An enhancement to these models is the multiscale model,^62^ where the macroscale behavior is modeled via a porous continuum model and a microscale representative volume element (RVE) is introduced, in which the coupled thermo-chemo-mechanical behavior is explicitly resolved. The RVE is selected to be large enough to capture the key statistical features of the microstructure yet small enough to function as a repeatable unit cell for upscaling. For coupled transport or fluid–solid interaction studies, the RVE can explicitly include pore space and air, allowing the model to resolve local flow fields, pressure gradients, and convective/diffusive transport around particles. An example of such a model is shown in Figure 3D, where the reactor bed temperature lift is computed through a porous continuum model coupled to the local behavior of the reaction front in a particle which varies along the bed length. While more computationally expensive, this approach benefits from increased fidelity in understanding transport phenomena at different spatial and times scales within TES materials. Finally, discrete particle models can be used when local microstructure, contact mechanics, and particle rearrangements are critical. In such an approach, computational fluid dynamics (CFD) is coupled with discrete element methods (DEM) to resolve gas flow around and through a collection of individually tracked particles. This is valuable in simulating processes where heterogeneity is critical, such as in fluidized beds, with the main drawback being that it is computationally expensive.^63^

## Composite Materials Design

*In situ* properties characterization and coupled continuum models are critical for predicting degradation mechanisms in salt hydrates. As discussed previously, however, pristine salt hydrates often suffer from hygrothermal and mechanical instabilities during charge/discharge cycling. The ideal TCM must satisfy a challenging set of simultaneous property constraints (Figure 1A) that no single material can achieve. To address this, composite materials can be engineered by combining components that enhance mechanical stability and hygrothermal stability, while also designing for high storage capacity and enhanced thermal transport. The architecture of the composite plays a crucial role in defining the mechanical strength, morphology, and transport, thereby shaping the overall storage performance and cycling stability. Significant efforts have been made by fabricating different composite materials, but a holistic design framework to achieve the desired properties is lacking. This is where the coupled continuum models discussed in the previous section can be leveraged to guide the development of new composite material architectures with optimal thermo-chemo-mechanical behavior.

Composite materials can be engineered by combining components that enhance mechanical stability and hygrothermal stability, while also designing for high storage capacity and enhanced thermal transport.

### Mechanical Stability

Mechanical stabilization of salt hydrates can be achieved through the addition of a polymer to form a composite. The polymer can serve as an encapsulating matrix or binder by leveraging its flexible nature to accommodate volumetric changes and fracture, thereby improving the cyclability of salt hydrate TCM composites.^64^ As a matrix, polymers are typically used for encapsulation, which can be broadly divided into two categories: core–shell^65^ and gelation syntheses.^66^ For core–shell encapsulation, a core- or shell-first method may be considered. In the core-first method, the salt is the base (core) around which the polymer shell is formed, such as coating polymers around K_2_CO_3_ beads.^65^ A shell-first method has been developed using mesoporous hollow silica spheres that are loaded with salt,^67^ but this has not been demonstrated with a polymeric matrix (to the best of our knowledge) and provides an opportunity for developing new encapsulation techniques. While the synthetic route for these two core–shell encapsulation matrices differs, the resulting composite includes a matrix that exists as a spherical shell around a salt hydrate core. This allows for salt particles to expand during hydration against the compliant polymer matrix, thereby limiting strain and reducing mechanical degradation in the form of fracture.^65^ Gelation synthesis results in a different kind of encapsulation where the matrix is formed both around and intertwined with the salt, as illustrated in Figure 4A. For example, Kallenberger et al. synthesized a biopolymer hydrogel alginate matrix with embedded salt hydrates by reacting divalent cations (Ca^2+^ and Sr^2+^) with sodium alginate.^66^ The matrix is effective for enhancing mechanical stability as hydrogels can accommodate water absorption by expansion of their polymer network when paired with sorbent materials like salt hydrates.^68^ Regardless of the encapsulation architecture, the polymer should be selected such that it is vapor permeable in order to enhance mechanical stability without inhibiting mass diffusion and reaction kinetics.

**Figure 4:**
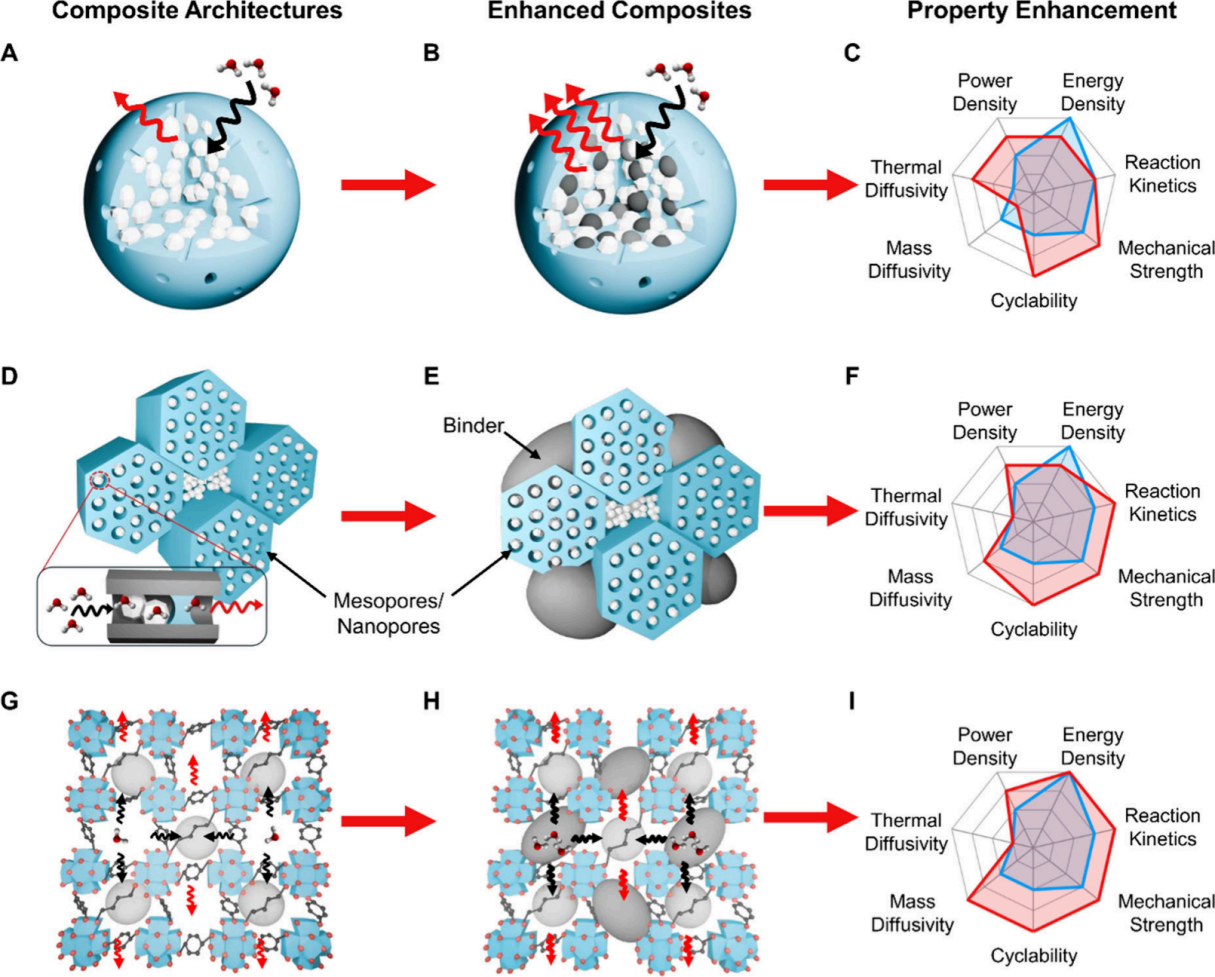
Composite architectures for salt hydrate TCMs to mitigate mechanical and hygrothermal degradation: (A) encapsulation of salt within a polymer matrix with macropores, (D) soft-templated impregnation of salt within silica mesopores, and (G) impregnation of salt within metal–organic framework micro- and mesopores. These composites can be further enhanced by adding (B) thermal fillers and (E, H) polymeric binders. (C, F, I) Composite materials (red polygon) can address the property limitation associated with pristine salts (blue polygon).

Polymers can be used as binders to enhance adhesion between salt particles, which also contributes to mechanical stability. Binders that have been used in electrochemical batteries, such as poly­(ethylene oxide) (PEO), poly­(acrylic acid) (PAA), and sodium carboxymethyl cellulose (Na-CMC), can also be used to enhance the stability of composite TCMs. These binders can improve TCM structures via crack-bridging, load sharing, and strengthening particle–matrix adhesion, thereby redistributing incompatibility stresses that arise from the nonuniform reaction-driven deformation discussed earlier. Polymeric binders can increase fracture toughness and fracture energy via polymer pull-out/elongation, viscoelastic energy dissipation, and improved interfacial adhesion.^69^ The amount of binder (relative to the salt) should be selected such that it increases mechanical integrity while simultaneously maintaining high energy storage density.

Polymer encapsulants and binders are inactive components within the composite as they do not participate in the thermochemical reaction. As such, their content should be carefully optimized so as not to hinder vapor/heat transport or storage density while providing the level of mechanical integrity required. This is where *in situ* measurements can be leveraged to provide time-resolved maps of phase transformations, mass and heat flow, and morphology (porosity and crack formation) evolution within the composite during cycling with the goal of keeping reaction rates and overall cycling performance stable. Additionally, computational models can be used for cost-effective exploration of candidate polymers tailored to a given salt, while CFD correlated simulations would allow for flow visulaization and prediction of transport pathways as a function of cycling.

### Hygrothermal Stability and Storage Capacity

Composites can also be engineered to hygrothermally stabilize salt hydrates while contributing to energy storage capacity, unlike the inactive polymer matrices described in the previous section. Specifically, matrices such as silica, zeolites, and metal–organic frameworks (MOFs) have dual functionality as they can stabilize salt hydrates via impregnation and adsorb water, which contributes to the storage capacity.^70–72^ These matrices have large pore volumes and surface areas, which improve the water vapor diffusion and reaction kinetics.^72,73^ Additionally, mesoporous matrices can prevent salt leakage after deliquescence via capillary attraction within the nanopores (nanoconfinement), which addresses hygrothermal instabilities during cycling.^74^ In wet impregnation, salt is loaded via its dissolution in a solvent (e.g., methanol or water), mixing with the matrix, and subsequent drying to precipitate salt within the pores of the matrix. Various modifications of this synthesis process exist, such as introducing high temperatures or vacuum to control the amount and distribution of salt within the pores. Dry impregnation is not a solventless synthesis, but instead refers to utilizing a solvent volume nearly identical to the pore volume of the matrix.^75^

Silica has been widely investigated as a physical sorbent, where its hydroxyl groups serve as a site for water adsorption.^76^ The sorption performance of silica can be significantly improved with the addition of salt hydrate TCMs; incorporating CaCl_2_ into mesoporous silica (7.5 nm pores) increased sorption capacity from 0.1 g/g to 0.75 g/g.^77^ Silica composites have enhanced hygrothermal stability as the impregnated salt is confined within the matrix mesopores (<50 nm) that prevent the salt solution from leaking and agglomerating (Figure 4D). Recent literature has shown that these capillary forces are amplified with smaller and more ordered pores (<10 nm), yielding stable composites with CaCl_2_ even at high vapor pressures.^78^ In order to further enhance silica composites, polymeric binders can be used; this adds mechanical stability to the hygrothermal stability of silica for increased reaction kinetics, power density, mechanical strength, and ultimately cyclability (Figure 4E, F).

Zeolites have been investigated as another class of matrix materials given their crystalline microporous nature and sorption ability due to well-defined pores and interconnected channel networks. Salt impregnation has been investigated as a strategy to enhance their sorption capacity, and in several cases, superior performance has been reported.^79–81^ However, deliquescence of the impregnated salt may induce changes in pore size, compromising the role of the zeolite as a stable and confined backbone structure. Consequently, the performance of salt-zeolite composites is strongly dependent on pore size, salt loading, and zeolite type, all of which must be carefully optimized to achieve reliable and efficient sorption behavior.^38,82^

More recently, MOFs have emerged as promising matrix materials given their crystalline and tunable porous structures with high surface areas.^83^ Their sorption performance can be tailored through functional groups, such as amino moieties on the organic linkers or hydroxyl groups on metal nodes, which modulate both the total uptake and the location of the adsorption step. As with silica and zeolites, salt impregnation has been investigated as a strategy to enhance the sorption capacity of MOFs.^84^ Salt nanoconfinement within MOF pores (Figure 4G) can also mitigate hygrothermal challenges by preventing leakage, stabilizing the salt phase, and preserving structural integrity during repeated hydration-dehydration cycles. The result is a composite that effectively combines the high energy storage density of salts with the structural stability and sorption kinetics of MOFs, as demonstrated by Permyakova et al.^85^ When hydrophilic MOFs are combined with polar salts, their hydrophilicity increases, making water desorption difficult. In this case, overall water uptake may not rise significantly with higher salt loading, as MOF physisorption dominates.^85^ However, careful selection of the type of MOF is necessary to achieve enhanced performance for thermal energy storage. When hydrophilic MOFs are combined with polar salts, their hydrophilicity increases, making water desorption difficult. In this case, overall water uptake may not rise significantly with higher salt loading, as MOF physisorption dominates.^85^ Moreover, hydrophilic MOFs with 1D pore channels, when fully loaded with salt, can create diffusion barriers that hinder water desorption.^85^ In contrast, amphiphilic MOFs enable efficient water absorption and desorption, while promoting a synergistic interaction between salt chemical reaction and MOF physisorption. This helps maximize water uptake and energy storage density, making amphiphilic MOFs an attractive choice for future MOF-salt composite design. Building on these insights, future research should focus on systematic evaluation of MOFs with varying pore sizes, polarities, and frameworks to identify robust matrices for TCM impregnation. MOFs can also be paired with polymeric binders, thus incorporating the energy and power density, reaction kinetics, and mass diffusivity of MOFs with the mechanical strength and adhesive properties of polymers (Figure 4H, I).

In all these impregnated composites, interfacial chemo-mechanical mechanisms play an important role, which can be explored using computational and *in situ* methods. These tools can be used to track salt distribution, phase transitions, and pore-filling dynamics, thereby directly linking transport properties and energy/power density to stress accumulation and cracking. These insights can reveal optimal pore-size and loading regimes that maximize water uptake and kinetics while minimizing mechanical damage.

### Salt Loading and Thermal Conductivity

Beyond the matrix architecture (encapsulation, binder, or impregnation), careful optimization of the salt loading (mass fraction of salt in the composite) is critical, as it directly impacts energy density and cyclability. A high loading can cause deliquescence and salt leakage during hydration, while a low loading will limit the energy density. Polymer encapsulant matrices have high salt loading potential, with gelation-based alginate hydrogels reported to have 80–90 wt % salt.^66^ However, with expanded graphite incorporated to enhance the thermal conductivity, a similar alginate matrix showed significant leakage at loadings above ∼65 wt %.^86^ This suggests that the salt loading should be varied for stability while ensuring minimal reduction in energy density. For impregnated matrices like silica, salt will crystallize and agglomerate on the composite outer surface at high loadings which no longer benefits from nanoconfinement.^87^ Additionally, impregnated MOFs and zeolites may experience vapor diffusion resistance or decreased sorption capacity at high loadings due to pore blockages.^23,85^

Finally, the inherently low thermal conductivity of salt hydrates and the aforementioned matrix materials (∼0.4–2 W/m-K) significantly limits heat addition/extraction and consequently the power density.^36,88,89^ It is thus necessary to design composites not only for high energy storage density and mechanical stability, but for thermal transport as well. To address this, thermally conductive materials based on carbon have emerged as effective fillers for salt hydrated salts, owing to their high thermal conductivity, low density, and tunable surface chemistry. Incorporation strategies include the carbon additive method, a cost-effective physical blending approach with low filler loading, and the carbon skeleton method, where salts are impregnated into porous carbon frameworks to achieve superior thermophysical performance. Carbon nanotubes (CNTs)^90–92^ and expanded graphite^93,94^ are particularly promising due to their intrinsic conductivity and ability to form percolation networks for heat transport through the composite. Depending on the dimensionality and geometry of the filler, different percolation models can be utilized to predict optimal volume fractions within the composite.^95–97^ One-dimensional fillers such as CNTs, with their high aspect ratios, can establish percolated thermal pathways even at low loadings, improving performance without compromising on storage capacity or kinetics. Percolation models paired with interfacial thermal resistance models can provide a deeper mechanistic understanding of filler–matrix interactions to achieve improved effective thermal conductivity values in composites. This will enable composites (Figure 4B) where macroporous encapsulate architectures and thermal fillers are paired to improve both mechanical stability and cyclability with enhanced thermal diffusivity and power density.

## Summary and Outlook

Energy storage based on thermochemical salt hydrates is promising for cost-effective and long duration storage. However, its successful implementation is predicated on advancing the fundamental knowledge of the underlying coupled thermo-chemo-mechanics that dictate cycling stability in these systems. Over the past decade, significant research has been done on TCMs, ranging from materials synthesis to reactor designs to HVAC integration.^18,19,98,99^ However, there is a disconnect between the material degradation mechanisms and system design, which often manifests as low performance and short lifetimes under realistic operating conditions.

This perspective lays out the experimental and theoretical framework that is necessary to achieve high-performance TCM-based energy storage. Given the coupled thermo-chemo-mechanical behavior of these materials, standard characterization techniques are presented for measuring thermochemical properties (energy density and reaction kinetics) of different salt hydrates. Specifically, TGA/DSC experiments with an integrated humidity generator should be used to screen materials for long-term cyclability. Small-scale samples (∼5 mg) with minimal vapor diffusion resistance can be used to provide insight into reaction kinetics. Additionally, thermophysical and transport properties (thermal conductivity and mass diffusion coefficient) must be characterized in relevant form factors. Pellets (∼1–5 mm) are the preferred form factor to reduce pressure drop within a packed bed reactor. In addition to heat and mass transport, mechanical degradation during repeated hydration/dehydration of these pellets must also be characterized. *Ex situ* methods provide only the “before” and “after” states, often missing the evolution of reaction fronts, stress localization, and phase instability, all of which play a critical role in driving mechanical degradation. To address this, *in situ* characterization techniques are outlined that can provide insight into the link between structural transitions, transport, and stress generation under realistic operating conditions. Building on these material-level characterization efforts, adopting a “unit-cell” reactor geometry (e.g., representative pellet or modular packed bed) would help establish a common set of outputs (usable energy storage density, power density, and temperature lift) akin to Ragone and rate capability plots in electrochemical systems. Taken together, these efforts can provide insight into the following research questions:

1. What are the mesoscale mechanisms that contribute to structural and mechanical instability (degradation) during transformation processes?
2. What are the dynamic and coupled interactions between the solid and fluid in representative heterogeneous environments that impact reactor performance?

These material properties also feed into continuum models that can be used to predict degradation induced by coupled thermo-chemo-mechanical processes to significantly reduce experimental iterations. The theoretical framework must explicitly couple mechanical degradation with hygrothermal transport and reaction kinetics to resolve evolving temperature/concentration (or reaction-extent) fields and reaction front propagation, which in turn promote cracking/fracture. Since cycling produces large mechanical stresses and damage, models should include constitutive and failure descriptions that link stress states to observed damage modes. These models should be used to provide insight into the following research questions:

3. What are the appropriate continuum kinematics descriptors for the thermo-chemically induced transformations in salt-hydrates?
4. What are the phenomenological constitutive equations that couple gradient-induced mechanical stress (instabilities) with transport in these materials during charge–discharge?

With the knowledge obtained from characterization and continuum models, high-performance composite materials can be designed with minimal thermo-chemo-mechanical degradation. Encapsulation and impregnation-based synthesis techniques are outlined to obtain salt-in-matrix composite materials with hygrothermal stability. Additionally, active matrix materials are discussed that contribute to energy density, while binders and thermal fillers can enhance mechanical stability and thermal conductivity of these composites over thousands of cycles. Research should focus on addressing the question:

5. What composite architectures can enable thermo-chemo-mechanical resilience by minimizing irreversibility and maximizing storage capacity during cyclic transformations?

Transforming thermochemical materials into viable energy storage technologies thus requires a concerted and interdisciplinary effort integrating materials design, advanced experimental characterization, and predictive multiscale modeling to bridge chemistry, mechanics, and reactor engineering.
